# Long term proliferation and physiological response of embryogenic callus in Slash pine (*Pinus Elliottii* Engelm)

**DOI:** 10.1038/s41598-025-06436-5

**Published:** 2025-07-01

**Authors:** Zishan Cheng, Cangfu Jin, Min Yi, Jinwen Xie, Meng Lai, Xiujing Xiao, Lu Zhang

**Affiliations:** Jiangxi Province Key Laboratory of Subtropical Forest Resources Cultivation, Co-innovation Center of Jiangxi Typical trees Cultivation and Utilization, Jiangxi Agricultural University, Nanchang, 2011, 330045 Jiangxi China

**Keywords:** *Pinus elliottii*, Somatic embryogenesis, Long-time proliferation, Embryogenic callus, Physiological indicator, Biotechnology, Physiology

## Abstract

Slash pine (*Pinus elliottii* Engelm.) is an economically significant tree species valued for its timber and resin production. Establishing a somatic embryogenesis system is essential for the effective utilization of slash pine. This study aims to develop an optimized cultivation system for the long-term proliferation of embryogenic callus (EC) in slash pine. The study used the EC induced from immature zygotic embryos as materials to investigated the effects of plant growth regulators (PGRs), subculture cycles, and subculture durations on the EC proliferation coefficient and the number of somatic embryos (SE). Additionally, we monitored the changes in the proliferation coefficient, the number of SE, and physiological and biochemical indicators of EC during long-term proliferation. The EC proliferation coefficient reached its highest value (7.65) when the DCR medium was supplemented with 1.0 mg/L 2,4-dichlorophenoxyacetic acid (2,4-D) and 1.0 mg/L 6-benzylaminopurine (6-BA). Refreshing the culture medium every 14 days, EC maintaining proliferation coefficient (4.05) and embryogenic potential for approximately three year (980 d), but the number of somatic embryos was low and significantly declined after 140 days.The additional supplementation of 1.0 mg/L 2- (1-naphthyl) acetic acid (NAA) to the original proliferation medium further enhanced SE induction, achieving a maximum of 143.25 SE per gram of EC. Under the optimal proliferation culture conditions, EC was subcultured for 392 days, with no significant differences observed in the proliferation coefficient and the number of SE. The results of correlation and path analyses between physiological indicators and proliferation coefficient indicate that the proliferation coefficient of EC is mainly related to the content of storage substances (starch and soluble sugars) and the activities of redox-related enzymes (POD and CAT). The optimized long-term proliferation culture system for EC in slash pine provides an efficient approach to enhance the production of EC and SE.

**Supplementary Information:**

The online version contains supplementary material available at 10.1038/s41598-025-06436-5.

## Introduction

Slash pine (*Pinus elliottii* Engelm.) an economically valuable tree species, and is characterized by strong adaptability, high resistance, rapid growth, and high resin yield, it native to the wetlands and coastal regions of the southeastern United States, now it has been widely introduced to various parts of the world and plays a significant role in global forestry production^1,2^. And it was introduced to China in the early 20th century, and has become an important economic tree species in Jiangxi Province^3^. However, traditional breeding methods face challenges such as long breeding cycles and the offspring of hybrid breeding have genetic instability, which hinder their ability to meet the demands of large-scale production. Somatic embryogenesis is a tissue culture technique that induces somatic cells to re-enter the cell cycle and generate new embryos, similar to zygotic embryogenesis without fertilizatio^4,5^. Compared to conventional propagation, somatic embryogenesis enables faster production of new plants for research and commercial applications and is considered a viable approach to address the supply-demand imbalance of elite species^5,6^.

Since the first successful application of somatic embryos on *Picea abies* by Hakman et al.^7^ in 1985, this technology has been extended to various conifer species including *Abies nebrodensis*^8^*Pinus koraiensis*^9,10^*Picea glauca*^11^*Larix decidua*^12^*Abies nordmanniana*^4^*Pinus pinaster*^13^. Furthermore, somatic embryogenesis technology system of slash pine is also being actively explored. In 1989, Jain et al.^14^ initiated the study of somatic embryogenesis in slash pine by using immature zygotic embryos as experimental material, successfully inducing embryogenic callus (EC) and then producing somatic embryos (SE). Subsequently, Hu et al.^5^Liao and Amerson^15^Newton et al.^16^and Tang et al.^17^ obtained regenerated plants of slash pine. Additionally, Yang et al.6 found that the adding 5 mg/L abscisic acid (ABA) to the suspension proliferation culture medium significantly increased SE yield. Despite these advancements, large-scale SE production in slash pine remains challenging due to the induction rate of EC in slash pine can only reach a maximum of 33%^18^, and the longest duration for maintaining EC is 6 months. Additionally, the differentiation efficiency of EC into SE is limited, and the proliferation capacity and embryogenic potential of EC are difficult to sustain over the long term^19,20^.

The embryonic maintenance and somatic embryo yield of EC is an important evaluation index in the large-scale breeding of conifer SE^21^. Establishing an efficient and stable long-term proliferation system for EC is a key step in achieving the industrialized production of somatic embryos^22,23^. For example, yield and quality of mature somatic embryos in *Pinus pinaster*, is strongly dependent on the EC line^24^. However, long-term subculture often leads to a decline in embryogenic potential and abnormal morphological changes in EC, posing a major challenge for sustained SE production^25–27^. Studies indicate that optimizing culture conditions, such as the types and concentrations of plant growth regulators (PGRs), culture cycle, and sucrose concentration, can enhance EC proliferation efficiency and maintain embryogenic capacity^28–30^. For instance, in *Picea koraiensis*, a combination of 0.21 mg/L BA, 0.20 mg/L KT, 1.85 mg/L NAA, and 20 g/L sucrose significantly improved EC proliferation efficiency^31^. In *Pinus taeda*, media supplemented with 0.1 mg/L 2,4-D, 0.05 mg/L BA, and 0.05 mg/L KT, or 0.5 mg/L NAA, 0.05 mg/L BA, and 0.05 mg/L KT, proved effective for EC maintenance^26^.

Somatic embryogenesis is a complex process that involves energy storage and the cooperative action of antioxidant enzymes, which together regulate EC proliferation as well as the formation and development of SE^32^. Storage compounds, such as starch, soluble sugars, and soluble proteins, are fundamental to cellular metabolism, and reactive oxygen species and redox homeostasis are also crucial for EC proliferation and differentiation^33^. Investigating the physiological mechanisms underlying somatic embryogenesis is essential for the precise regulation of this process. For example, phytosulfokine has been shown to maintain reactive oxygen species homeostasis by reducing peroxidase activity during early SE induction, thereby promoting SE formation in *Cunninghamia lanceolata*^34^and phytosulfokine has been demonstrated to facilitate the transition of embryogenic masse from pro-embryogenic mass I(PEMI)to PEMII or PEMIII stages in *Pinus massoniana*, and increased the accumulation of soluble sugars, soluble proteins, and starch^35^. In slash pine, significant variations in soluble proteins, soluble sugars, starch content, and antioxidant enzyme activities (POD, SOD, CAT) have been observed among EC cell lines, though no clear correlation with embryogenic potential has been established^36^. The physiological and biochemical responses of slash pine EC during long-term proliferation remain poorly understood, representing a critical knowledge gap.

This study addresses this gap by optimized long-term EC proliferation system for slash pine, examining the effects of PGRs, subculture cycles, and monitoring the changes in metabolite content indicators (soluble sugars, starch, soluble proteins), antioxidant enzyme activity indicators (SOD, POD, and CAT) on EC proliferation and SE production. The findings of this study are expected to provide a significant technical and theoretical foundations for the somatic embryogenesis of slash pine.

## Materials and methods

### Materials

The following experiments used EC as the material. EC were induced from immature zygotic embryo. Slash pine seeds were collected in mid-July from Baiyunshan Forest Farm, Ji’an City, Jiangxi Province, China. The seeds were sterilized by soaking in 75% (v/v) alcohol for 5 min, then rinsed with sterile water. The immature zygotic embryos were then inoculated onto the induction medium and cultured in the dark at 23 ± 1℃ for 60 days. The induction, proliferation and maturation of EC were conducted using the medium previously optimized by our research group^37^. All experimental materials used were embryogenic callus with stable subculture for 70 days in good condition.

### Methods

#### Culture conditions

**Induction culture** The induction medium is DCR basic medium^37^and supplemented with 2.0 mg/L 2, 4-D, 2.0 mg/L 6-BA, 2.0 mg/L NAA, 30 g/L sucrose, 500 mg/L CH, 500 mg/L L-glutamine, 250 mg/L MES and 4 g/L gellan gum. The pH is between 5.8 ~ 6.0^19,38^.

**Proliferation culture** The proliferation medium is DCR basic medium supplemented with 30 g/L sucrose, 4 g/L gellan gum, 500 mg/L L-glutamine and 500 mg/L casein hydrolysate. The pH was maintained between 5.8 and 6.0. The EC was cultured in the dark at a temperature of 23 ± 1℃^38^. Each dish was inoculated with two 1 g clumps of healthy EC that had white, filamentous outgrowths. The weight of the EC was measured after 14 days, and all cultures were placed onto new medium every 14 days.

**Maturation culture** The EC was transferred to DCR proliferation medium with no hormone supplementation and cultured for 10 days. Subsequently, the EC was transferred to a maturation medium, 1 g EC was evenly distributed across four dishes, with 0.25 g allocated to each dish. The maturation medium composed of DCR basic medium supplemented with 10 mg/L ABA, 100 mg/L polyethylene glycol 8000, 20 g/L maltose, 4 g/L gellan gum, 500 mg/L L-glutamine, 500 mg/L casein hydrolysate and 250 mg/L 2-Morpholinoethanesulphonic acid^37^. The pH was maintained 6.0. The culture was maintained in the dark for 60 days at 25 ± 1 ℃.

#### Experimental design

Experiment 1: Optimization of EC proliferation culture conditions

**Culture cycle of EC** The EC induced in 2021 was used as the experimental material. The EC were cultured on a proliferation medium supplemented with 2.0 mg/L 2,4-D and 2.0 mg/L 6-BA, which is the hormone combination with the highest proliferation efficiency obtained from the previous laboratory research^37^. The durations respectively are 4, 6, 8, 10, 12, 14, 16, 18, and 20 days. At each stage, the weight of the EC was measured to calculate the proliferation coefficient (Eq. 1) and the proliferation rate (Eq. 2). A single-factor experimental design was employed, with 1 g EC per dish, 6 dishes per treatment. The treatment was repeated three times.

1 $$\:\text{P}\text{r}\text{o}\text{l}\text{i}\text{f}\text{e}\text{r}\text{a}\text{t}\text{i}\text{o}\text{n}\:\text{c}\text{o}\text{e}\text{f}\text{f}\text{i}\text{c}\text{i}\text{e}\text{n}\text{t}=\frac{\:\text{E}\text{C}\:\text{w}\text{e}\text{i}\text{g}\text{h}\text{t}\:\text{a}\text{f}\text{t}\text{e}\text{r}\:\text{c}\text{u}\text{l}\text{t}\text{u}\text{r}\text{e}\:-\:\text{E}\text{C}\hspace{0.17em}\text{w}\text{e}\text{i}\text{g}\text{h}\text{t}\:\text{b}\text{e}\text{f}\text{o}\text{r}\text{e}\:\text{c}\text{u}\text{l}\text{t}\text{u}\text{r}\text{e}}{\text{E}\text{C}\:\text{w}\text{e}\text{i}\text{g}\text{h}\text{t}\:\text{b}\text{e}\text{f}\text{o}\text{r}\text{e}\:\text{c}\text{u}\text{l}\text{t}\text{u}\text{r}\text{e}}$$

2 $$\:\text{P}\text{r}\text{o}\text{l}\text{i}\text{f}\text{e}\text{r}\text{a}\text{t}\text{i}\text{o}\text{n}\:\text{r}\text{a}\text{t}\text{e}=\frac{\:\text{E}\text{C}\:\text{w}\text{e}\text{i}\text{g}\text{h}\text{t}\:\text{o}\text{n}\:\text{d}\text{a}\text{y}\:\text{n}+2\:-\:\text{E}\text{C}\:\text{w}\text{e}\text{i}\text{g}\text{h}\text{t}\:\text{o}\text{n}\:\text{d}\text{a}\text{y}\:\text{n}\:}{2}$$.

**Combination of PGRs concentrations** A completely randomized block design was employed using 0.5, 1.0, or 1.5 mg/L of 2,4-dichlorophenoxyacetic acid (2,4-D) and 0.5, 1.0, or 1.5 mg/L of 6-benzylaminopurine (6-BA), resulting in nine hormone concentration combinations. The fresh weight of the EC in each per dish was measured to calculate the EC proliferation coefficient (Eq. 1). Each treatment was replicated three times, and with 2 g EC per dish, 3 dishes per treatment.

Experiment 2: long-term maintenance and physiological and biochemical changes of EC

**Effects of subculture duration on EC proliferation coefficient and the number of SE** The EC induced in 2021 was used as the experimental material. The EC were cultured on a proliferation medium supplemented with 1.0 mg/L 2,4-D and 1.0 mg/L 6-BA. The EC proliferation coefficient (Eq. 1) was assessed on days 70, 140, 350, 700, and 980. Each treatment was replicated three times, and with 2 g EC per dish, three dishes per treatment.

After assessment, the EC after cultured 70, 140, 350, 700, and 980 days were cultured on a maturation medium respectively. After 60 days, the number of SE was assessed to calculate the number of SE induced by per gram EC (Eq. 3). Each treatment was replicated three times, with 0.25 g per dish, four dishes per treatment.

3 $$\:\text{T}\text{h}\text{e}\:\text{n}\text{u}\text{m}\text{b}\text{e}\text{r}\:\text{o}\text{f}\:\text{S}\text{E}\:\text{i}\text{n}\text{d}\text{u}\text{c}\text{e}\text{d}\:\text{b}\text{y}\:\text{p}\text{e}\text{r}\:\text{g}\text{r}\text{a}\text{m}\:\text{o}\text{f}\:\text{E}\text{C}\:\:=\frac{\text{N}\text{u}\text{m}\text{b}\text{e}\text{r}\:\text{o}\text{f}\:\text{S}\text{E}}{\text{E}\text{C}\:\text{w}\text{e}\text{i}\text{g}\text{h}\text{t}\:\:\text{b}\text{e}\text{f}\text{o}\text{r}\text{e}\:\text{c}\text{u}\text{l}\text{t}\text{u}\text{r}\text{e}\:\left(\text{g}\right)}\:$$.

**Measurement of physiological and biochemical indicators** After assessment, on the 70th, 140th, 350th, 700th, and 980th day of culture, 5 g EC was taken and transferred to cryovials respectively, immediately submerged in liquid nitrogen, and stored at −80 °C until further analysis.

Physiological indicators: Starch and soluble sugars contents were assessed using the sulfuric acid-anthrone method and measured at 620 nm with a spectrophotometer (GENESYS 18 UV-Visible Spectrophotometer, Thermo Scientific)^38^. Soluble proteins contents were quantified using the Coomassie Brilliant Blue G-250 method at 595 nm^39^.

Biochemical indicators: while catalase (CAT) activity was quantified using the Coomassie Brilliant Blue G-250 method using UV spectrophotometry at 240 nm^39^. The activities of superoxide dismutase (SOD) and peroxidase (POD) were analyzed using kits from Nanjing JianCheng Bioengineering Institute. The SOD sample solution was prepared by homogenizing 0.1 g of sample in 400 µL of phosphate buffer (pH 7.0). The POD sample solution was prepared by homogenizing 0.1 g of sample in 900 µL of phosphate buffer (pH 7.0). The homogenate was centrifuged at 10,000 rpm for 10 min at 4 °C, and the supernatant as retained for analysis.

Experiment 3: Maintenance of embryogenic capacity during long-term EC proliferation.

**Combination of PGRs types and concentrations** EC induced in 2023 was used as the experimental material. Based on the optimal combination of PGRs concentrations, this study explored the effects of six PGRs on EC proliferation: 2, 4-D, 6-BA, kinetin (KT), 2-(1-Naphthyl) acetic acid (NAA), abscisic acid (ABA), and brassinolide (BL) (Table 3). The weight of the EC was assessed after 14 days to calculate the proliferation coefficient (Eq. 1). Each treatment was replicated four times and with 2 g EC per dish, three dishes per treatment.

After assessment, the EC were cultured on a maturation medium. After 60 days, the number of SE was assessed to calculate the number of SE induced by EC per gram (Eq. 3). Each treatment was replicated three times, with 0.25 g per dish, four dishes per treatment.

**Effects of subculture duration on EC proliferation coefficient and the number of SE** EC were cultured on a proliferation medium supplemented with 1.0 mg/L 2,4-D, 1.0 mg/L 6-BA, and 1.0 mg/L NAA. The EC proliferation coefficient (Eq. 1) was assessed after subcultured for 112, 168, 224, 280, 336, and 392 days. Each treatment was replicated three times. 2 g EC per dish, three dishes per treatment.

After assessment, the EC were cultured on a maturation medium. After 60 days, the number of SE was assessed to calculate the number of SE induced by EC per gram (Eq. 3). Each treatment was replicated three times, with 0.25 g per dish, four dishes per treatment.

Additionally, the EC were stained with acid fuchsin (0.1%) and Evans blue (0.05%) double staining method, and observed under a MODEL ECLIPSE Ci-L microscope to assessed the structure of embryonal suspensor mass (ESM) after subcultured for 112, 280, 392 days.

### Statistical analysis

The data were analyzed using a one-way analysis of variance (ANOVA) with SPSS (v 25.0), followed by multiple comparisons using Duncan’s test. Path analysis was conducted to evaluate the effects of physiological indicators on proliferation coefficient. The model was developed using SPSS (v 25.0), and significant variables were identified through stepwise regression analysis, followed by the calculation of their path coefficients. Pearson’s correlation analysis, regression analysis, Dot-line plot, and histogram generation were performed using Origin Pro Learning Edition 2021. Figures modification using Adobe Illustrator. The data are expressed as mean values ± standard error.

## Results

### Culture cycle of EC

**Fig. 1:**
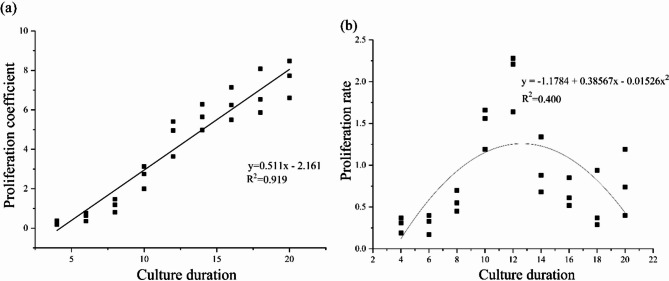
Culture cycle of EC (**a**) Linear regression analysis of culture duration on proliferation coefficient; (**b**) Quadratic regression analysis of culture duration on proliferation rate.

The experimental data indicate a significant positive correlation between proliferation coefficient and culture duration (Fig. 1a). Linear regression analysis produced the equation y = 0.511x − 2.161, with an R² value of 0.919, demonstrating a very high correlation between the two variables. As the culture duration increased, the proliferation coefficient also increased significantly. The fitted line closely matches the data trend, indicating that culture duration is a crucial factor affecting the proliferation coefficient.

As shown in the figure, the relationship between proliferation rate and culture duration follows a quadratic curve (Fig. 1b). The regression equation is y = −1.1784 + 0.38567x − 0.01526x², with a coefficient of determination R² = 0.400, indicating that culture duration accounts for 40% of the variation in proliferation rate. The results show that the proliferation rate peaks at around 12 days of culture, and then decreases gradually. Although some variability exists, the overall trend indicates an initial rise and subsequent decline in proliferation rate, highlighting the existence of an optimal culture duration.

**Fig. 2:**
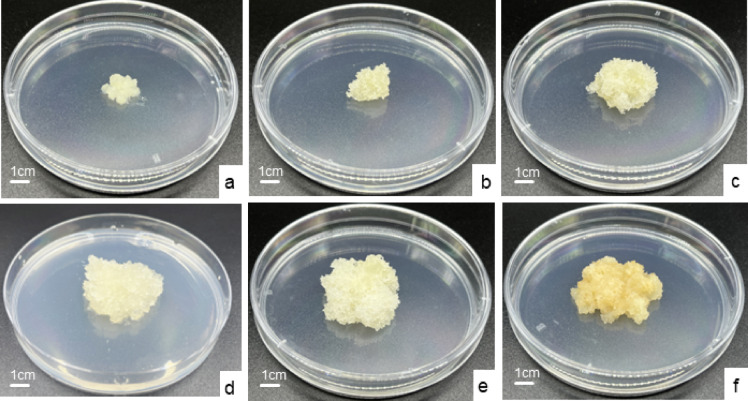
The EC growth status at different culture durations. (**a**) Day 1; (**b**) Day 4; (**c**) Day 12; (**d**) Day 14; (**e**) Day 16; (**f**) Day 20. All bars = 1 cm.

The EC growth status is shown in Fig. 2. EC was transferred to the proliferation medium (Fig. 2a). EC exhibited obvious growth on day 4 (Fig. 2b), characterized by translucent, filamentous protrusions. Rapid growth occurred between days 4 and 12, with the proliferation rate peaking on day 12 before declining (Fig. 2c). Notably, EC began to turn brown on day 14 (Fig. 2d). Dense filamentous protrusions were visible on the surface of EC on day 16, but no significant proliferation was observed in EC during days 14 to 16 (Fig. 2e). The internal tissue turned brown, the surface filamentous protrusions became inactive, and the callus structure became loose on day 20 (Fig. 2f). Therefore, the optimal subculture cycle for EC was determined to be 14 days, during which the EC exhibited rapid growth, high early embryogenic activity, and maximum proliferation coefficient.

### Effect of PGRs combinations on EC proliferation

Different combinations of 2,4-D and 6-BA concentrations had significant effects on the proliferation coefficient (Table 1) (*P* = 0.000). The results showed that Treatment 5 (1.0 mg/L 2,4-D and 1.0 mg/L 6-BA) achieved the highest proliferation coefficient (7.65), which was significantly higher than the other treatments. This was followed by Treatment 6 (1.5 mg/L 2,4-D and 1.0 mg/L 6-BA) with a proliferation coefficient of 6.39. The lowest proliferation coefficient was observed in Treatment 7 (0.5 mg/L 2,4-D and 1.5 mg/L 6-BA) at 3.65. Overall, the combination of 1.0 mg/L 2,4-D and 1.0 mg/L 6-BA significantly promoted proliferation and had the best effect. At this optimal concentration combination, the callus displayed a white, translucent appearance with characteristic filamentous structures (Supplementary Fig. S1).

**Table 1:** Effect of PGRs concentration combinations on EC proliferation coefficient.

Treatment	2,4-D mg/L	6-BA mg/L	Proliferation coefficient
1	0.5	0.5	5.54 ± 0.467bcd
2	1.0	0.5	5.87 ± 0.691abc
3	1.5	0.5	3.78 ± 0.861d
4	0.5	1.0	4.63 ± 0.721bcd
5	1.0	1.0	7.65 ± 0.565a
6	1.5	1.0	6.39 ± 0.179ab
7	0.5	1.5	3.65 ± 0.356d
8	1.0	1.5	5.31 ± 0.133bcd
9	1.5	1.5	4.41 ± 0.500 cd

Note: Different lowercase letters indicated significant differences among samples (Duncan test; *P* < 0.05).

### Effects of subculture duration on EC proliferation coefficient and the number of SE during long-term proliferation

**Fig. 3:**
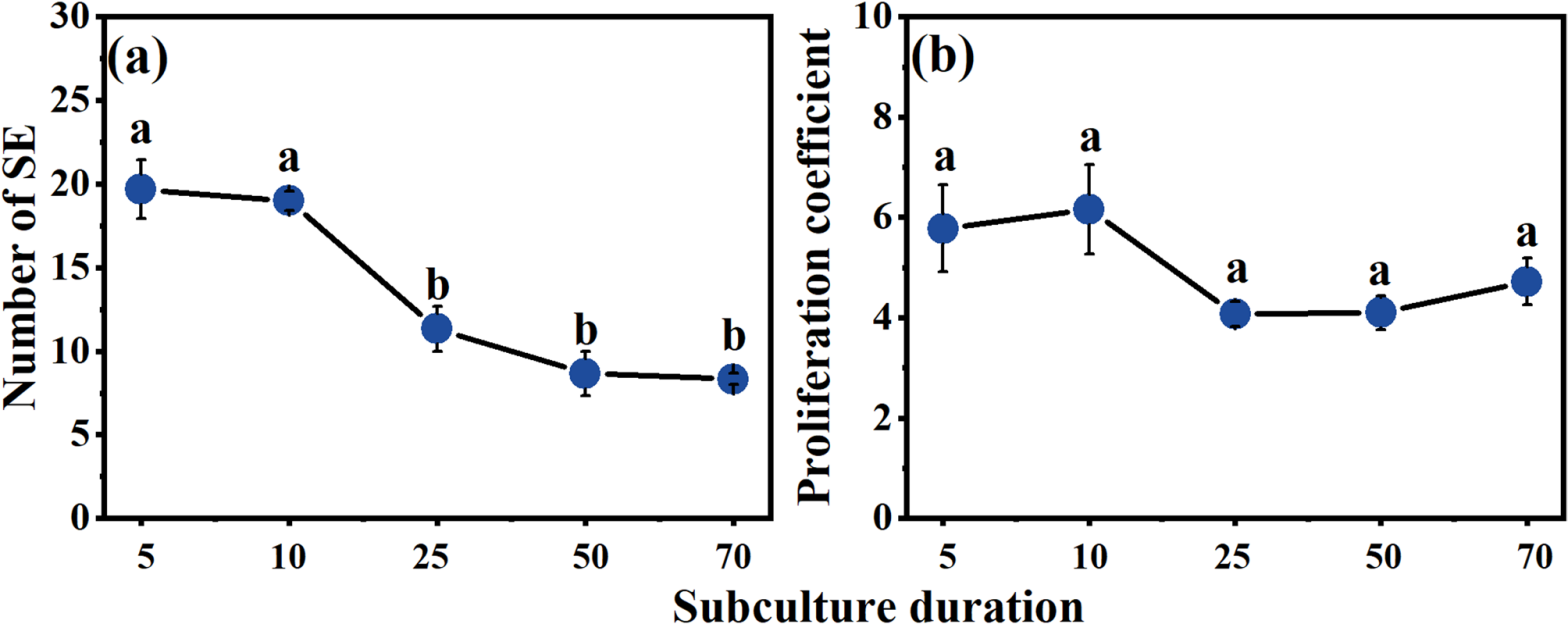
Effects of subculture duration on EC proliferation coefficient and the number of SE. (**a**) SE per gram of EC; (**b**) EC proliferation coefficient. Different lowercase letters indicated significant differences among samples (Duncan test; *P* < 0.05).

Subculture duration had no significant differences on EC proliferation coefficient (*P* = 0.121), despite the slight decrease in the EC proliferation coefficient with long-term subculture (Fig. 3b). The highest EC proliferation coefficient was observed at the 10th subculture, reaching 6.15. Although a decreasing trend in proliferation coefficient was noted after cultured 140 days, EC maintained a relatively high proliferation coefficient, achieving 4.72 at the 70th subculture. Subculture duration had a significant impact on the embryogenic capacity of EC (*P* = 0.000). The number of SE per gram of EC gradually decreased with long-time subculture, peaking at 19.67 during the 5th subculture (Fig. 3a). The results indicate that EC can proliferate stably for up to 980 days, while retaining its embryogenic capacity, under suitable conditions.

### The physiological response of EC during long-term proliferation

**Fig. 4:**
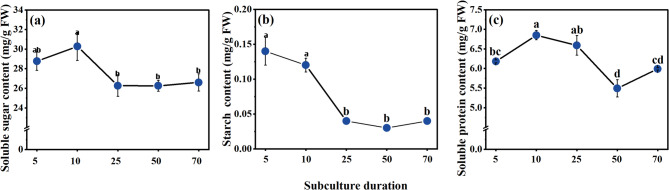
The changes in stored substances (soluble sugars, starch, soluble proteins) content during long-term proliferation. (a) Soluble sugars content; (b) Starch content; (c) Soluble proteins content. Different lowercase letters indicated significant differences among samples (Duncan test; *P* < 0.05).

**Fig. 5:**
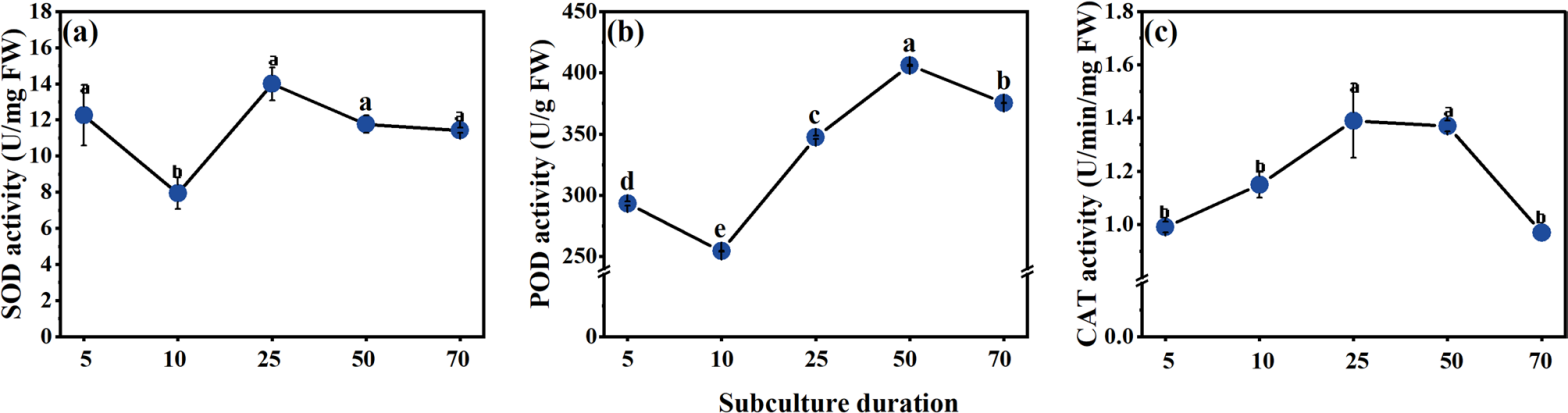
The changes in redox substances (SOD, POD, CAT) activity during long-term proliferation. (a) SOD activity; (b) POD activity; (c) CAT activity. Different lowercase letters indicated significant differences among samples (Duncan test; *P* < 0.05).

Physiological indicators change in EC after 5th, 10th, 25th, 50th, and 70th subculture is presented in Figs. 4 and 5. Significant differences were observed in stored substances (soluble sugars, starch, soluble proteins) content and redox substances (SOD, POD, CAT) activity in EC of different subculture times. The soluble protein and soluble sugars content of EC displayed a trend of initial increase followed by a decrease during long-term subculture. Both soluble proteins and soluble sugars content reached their peaks at the 10th subculture, with values of 6.85 mg/g and 30.28 mg/g, respectively. The soluble sugars content decreased to 26.8 mg/g at the 25th subculture and remained stable from the 25th to the 70th subculture. In contrast, soluble proteins content decreased to its lowest level of 5.49 mg/g at the 50th subculture. Starch content exhibited a significant decreasing trend throughout the long-term subculture process. It was highest at the 5th subculture, with a value of 0.14 mg/g, but decreased sharply at the 25th subculture and reached its lowest level of only 0.03 mg/g at the 50th subculture.

With increasing subculture times, CAT activity followed a trend of initial increase followed by a decrease, peaking at 1.39 U/min/mg FW at the 25th subculture. Both SOD and POD activities reached their lowest levels at the 10th subculture, with values of 7.94 U/mg FW and 254.33 U/g FW.

### The correlation analysis between proliferation coefficient and physiological changes

**Fig. 6:**
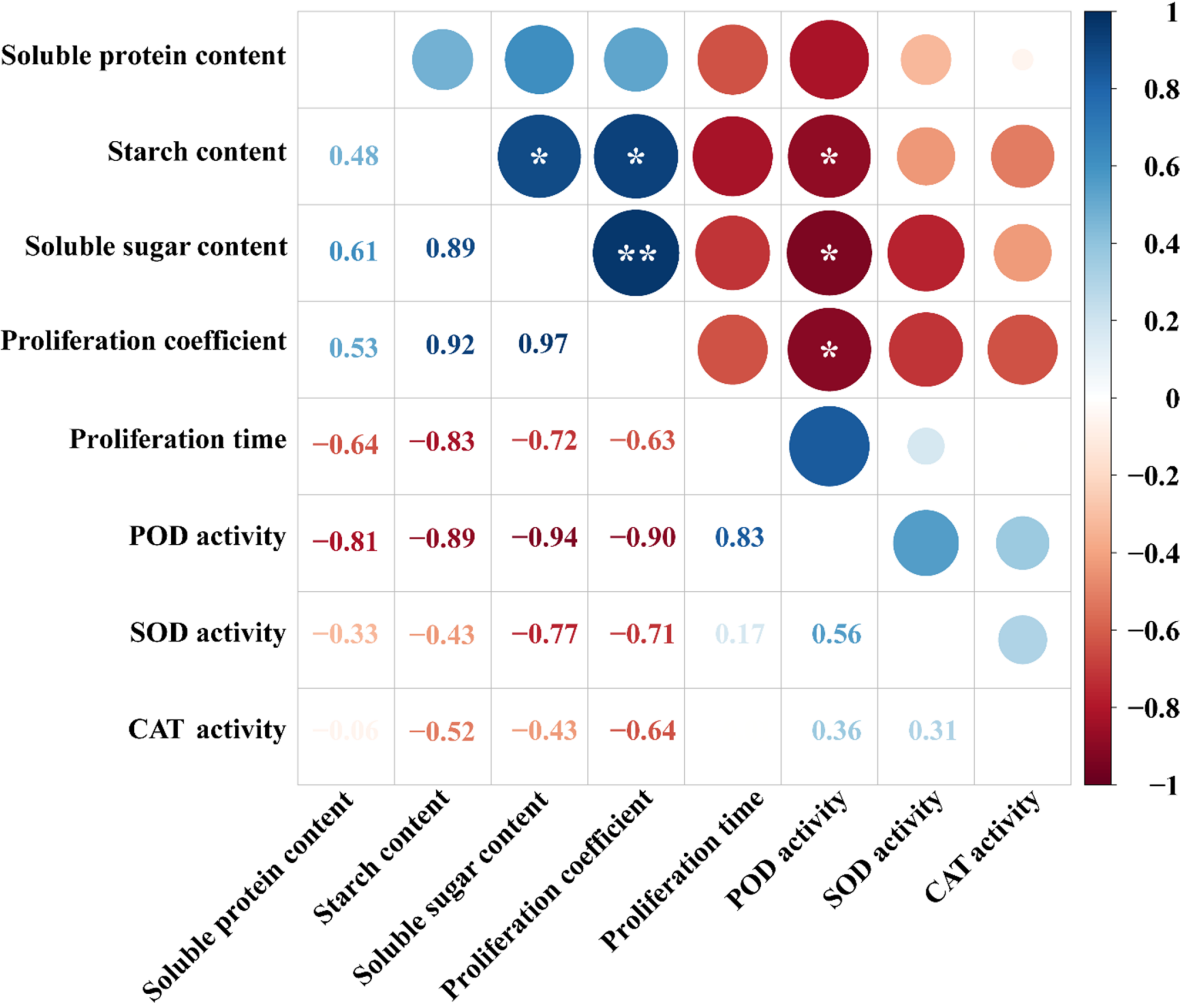
Correlation analysis between proliferation coefficient and physiological indicators of long-term proliferation of EC. Using Pearson correlation analysis, red represents a negative correlation, while blue represents a positive correlation. The closer the correlation coefficient is to 1, the stronger the correlation between the variables. * *P* ≤ 0.05; ** *P* ≤ 0.01.

Correlation analysis was conducted between the proliferation coefficient and physiological indices during the long-term proliferation of embryogenic callus (Fig. 6). The proliferation coefficient, starch content, and soluble sugar content were all negatively correlated with POD activity. In contrast, a positive correlation was identified between starch content and proliferation coefficient, as well as between soluble sugars content and starch content. Moreover, there was a significant positive correlation between soluble sugars content and proliferation coefficient(*P* < 0.01).These results indicate that the proliferation efficiency of embryogenic callus is mainly related to the content of storage substances (starch and soluble sugars) and the activity of redox substances (POD).

### Path analysis of the effect of physiological indicators on the proliferation coefficient

A path analysis with multiple stepwise regression was performed (Table 2), by using proliferation coefficient as the dependent variable and six physiological indicators as independent variables. The multiple stepwise regression equation was y = −5.972 + 0.451 X_1_ −1.3 X_2_ (R²=0.996), where X_1_ represent soluble sugars content and X_2_ represent CAT activity. The direct path coefficients were ranked in descending order as follows: CAT activity > soluble sugars content > starch content > SOD activity > soluble proteins content > POD activity. Notably, the sum of the indirect path coefficients for starch content (1.06) exceeded that of the other physiological indicators (−0.94 to 0.61). The multiple stepwise regression equation and path analysis indicated that soluble sugars content and CAT activity were the most significant physiological indicators directly influencing proliferation coefficient, while starch content had the largest indirect effect on proliferation coefficient.

**Table 2:** Path analysis of the effect of physiological indicators on the proliferation coefficient.

Physiological indicators	The direct path coefficients	The indirect path coefficients						The sum of the indirect path coefficients
		SOD activity	POD activity	CAT activity	Soluble proteins content	Starch content	Soluble sugars content	
SOD activity	0.06	0	−0.01	−0.40	0.01	−0.05	−0.35	−0.79
POD activity	−0.02	0.03	0	−0.47	0.02	−0.09	−0.42	−0.94
CAT activity	−1.30	0.02	−0.01	0	0	−0.05	−0.19	−0.24
Soluble proteins content	−0.02	−0.02	0.02	0.07	0	0.05	0.28	0.40
Starch content	0.10	−0.02	0.02	0.67	−0.01	0	0.40	1.06
Soluble sugars content	0.45	−0.04	0.02	0.56	−0.01	0.09	0	0.61

### Optimization of PGRs types and concentrations

The above research results show that we obtained the maximum number of somatic embryos was only 19.67 per gram on the 70th day, and it dropped sharply in the 25th generation during long-term proliferation. Therefore, different concentrations of combined hormones were added to optimize the existing culture conditions.

Different hormone combinations had significant effects on both proliferation coefficient (*P* = 0.00) and number of SE (*P* = 0.000). As shown in Table 3, the proliferation coefficients of Treatment 1 (3.94) and Treatment 2 (2.70) were relatively high, but both resulted in low numbers of SE (5 and 9.5, respectively). Treatment 3 showed the lowest values for both proliferation coefficient (0.59) and somatic embryo number (0.25). Although Treatments 4 and 5 had lower proliferation coefficients (1.49 and 1.06) compared to Treatment 1, they produced substantially higher numbers of somatic embryos, reaching 143.25 and 111.75, respectively. In summary, Treatment 4 yielded the highest number of somatic embryos while maintaining an acceptable proliferation coefficient, making it the optimal combination of plant growth regulators.

**Table 3:** Effect of combinations of PGRs concentration (mg/L) and types on EC proliferation coefficient and number of SE.

Treatment	2,4-D	6-BA	ABA	BL	NAA	KT	Proliferation coefficient	Number of SE
1	1.0	1.0	\	\	\	\	3.94 ± 0.22a	5 ± 1.47c
2	1.0	1.0	1.5	\	\	\	2.70 ± 0.35a	9.5 ± 2.87c
3	1.0	1.0	2.5	2.5	\	\	0.59 ± 0.20c	0.25 ± 0.25c
4	1.0	1.0	\	\	1.0	\	1.49 ± 0.20b	143.25 ± 12.36a
5	\	0.5	\	\	0.2	0.5	1.06 ± 0.08bc	111.75 ± 7.48b

Note: Different lowercase letters indicated significant differences among samples (Duncan test; *P* < 0.05).

### Effect of subculture duration on proliferation coefficient and number of SE

**Fig. 7:**
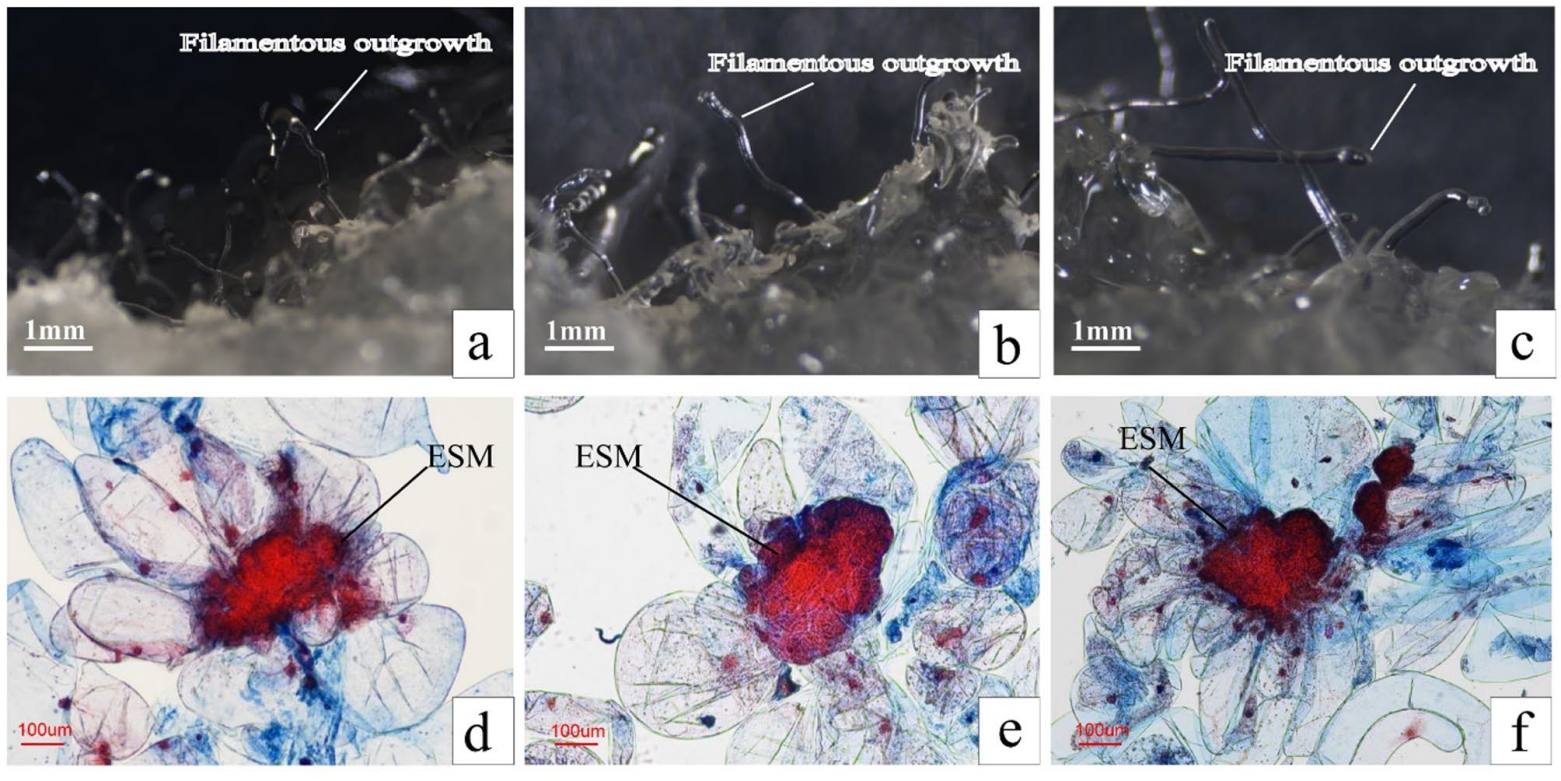
The filamentous outgrowths and the ESM structure of EC during long-term proliferation. a-c: The filamentous outgrowths of EC subcultured 112 days, 280 days, 392 days. (**a**): 112 days, (**b**): 280 days, (**c**): 392 days; The ESM structure of EC subcultured 112 days, 280 days, 392 days. (**d**): 112 days, (**e**): 280 days; (**f**): 392 days. All bars = 100 μm.

Under optimal conditions, the proliferation coefficient (*P* = 0.934) and number of SE (*P* = 0.159) showed no significant changes over many subcultures (Table 4). After 392 days of culture, the EC exhibited stable proliferation, with a proliferation coefficient of 1.5 and an average yield of 117 SE per gram of EC. The highest proliferation coefficient, reaching 1.53 times the initial value, was observed at 224 days, accompanied by an average of 123.33 SE per gram of EC. Notably, the maximum SE yield of 143.33 per gram of EC was achieved at 280 days of proliferation culture. Furthermore, Additionally, there were no significant changes in ESM of EC during the long-term subculture process, and EC remained embryogenic at 392 days (Fig. 7). EC induced from immature zygotic embryos (Fig. S2a) was successfully differentiated into SE (Fig. S2c, S2d) after long-term proliferation culture (Fig. S2b), further developing cotyledons (Fig. S2e) and forming complete regenerated plantlets (Fig. S2f). This study has achieved stable long-term proliferation culture of EC in slash pine.

**Table 4:** Effects of subculture duration on EC proliferation coefficient and number of SE.

Subculture duration (days)	Proliferation coefficient	Number of SE
112	1.36 ± 0.21a	105.67 ± 10.73a
168	1.50 ± 0.24a	106.67 ± 7.75a
224	1.53 ± 0.30a	123.33 ± 12.55a
280	1.27 ± 0.15a	143.33 ± 17.48a
336	1.29 ± 0.17a	104.33 ± 3.84a
392	1.50 ± 0.27a	117.00 ± 6.43a

Note: Different lowercase letters indicated significant differences among samples (Duncan test; *P* < 0.05).

## Discussion

### Long-term proliferation of EC

It is difficult for EC to maintain stable proliferation and embryogenic potential during long-term proliferation. Previous studies on slash pine^5^ and *Pinus koraiensis*^40–42^ have shown that EC declines after six months of subculture. But the embryogenic lines of *Larix × eurolepis*^43^and *Larix leptolepis*^44^ remained stable after four and nine years of subculture, respectively. It indicates that in some species, EC can proliferate indefinitely under suitable conditions^32,41,42^. EC maintaining a relatively stable proliferation coefficient (4.05) for approximately three years(980 d), but the number of somatic embryos was low and significantly declined after 140 days. This may be due to the high proliferation rate of embryogenic callus, which could inhibit the development of somatic embryos^45^but more evidence is still needed to confirm this connection. To increase somatic embryo yield, this study further optimized the concentrations of plant hormones, using NAA supplementation to enhance somatic embryo production. Although the proliferation coefficient of embryogenic callus decreased, the callus was able to stably proliferate and efficiently differentiate over 392 days of continuous subculture.

### The role of PGRs in EC proliferation and embryogenesis

Hormonal regulation plays a key role of EC proliferation and differentiation. The appropriate concentration, variety, and combination of plant growth regulators are crucial for SE and plant propagation^46^. Auxins and cytokinins are essential for maintaining optimal in vitro culture conditions^45,47^. Auxins like 2,4-D are critical for callus induction and proliferation, while cytokinins such as 6-BA promote cell division and differentiation^48,49^. The synergistic use of 2,4-D and 6-BA has been shown to enhance EC proliferation in various conifer species, such as *Pinus koraiensis*^29^ and *Pinus nigra*^50^. In this study, the effects of different PGR combinations on EC proliferation and embryogenic potential vary significantly. While the combination of 2,4-D and 6-BA achieved the highest proliferation coefficient, it resulted in a low number of SEs. It has been established that prolonged culture with 2,4-D reduces somatic embryo yield in sugarcane EC by increasing putrescine and spermidine levels, and interferes with the synthesis of key soluble proteins such as late embryogenesis abundant soluble proteins, chitinase, oleosin, and heat shock soluble protein^51,52^. These disruptions may negatively impact embryogenesis during long-term culture, but due to the fundamental differences in hormonal regulation between monocots and gymnosperms, further validation is needed in conifers.

In this study, the addition of other PGRs, such as ABA, BL, and NAA, resulted in a decline in proliferation coefficient, this may due to the disruption of hormonal balance, though the mechanisms remain unclear. NAA, commonly used to induce somatic embryogenesis, showed the highest SE yield in this study, with over 100 SEs per gram of EC. Similar results were observed in other species. For example, 3 mg/L NAA and 0.2 mg/L TDZ were most effective of *Schisandra chinensis* callus proliferation and metabolite synthesis^53^high NAA concentrations (10.0 mg/L) enhanced somatic embryogenesis in *Ranunculus sceleratus*^54^and 1 mg/L NAA and 0.25 mg/L 6-BA yielded the highest somatic embryo production at 166 embryos per gram in *Pinus koraiensis*^28^.

### The optimal subculture cycle of EC

Regular subculturing prevents the accumulation of toxic metabolic byproducts and ensures a stable hormonal and nutritional environment, both of which are critical for sustained EC proliferation. The optimal subculture cycle for EC varies among different species. This study determined that the optimal subculture cycle for EC in slash pine is 14 days. The optimal subculture interval for *Larix kaempferi*^55^ is 20 days, while for *Cinnamomum camphora*^56^it ranges from 20 to 25 days. In contrast, the callus of *Pinus koraiensis* requires transfer to fresh medium every 13 to 15 days to maintain vigorous activity and achieve higher proliferation efficiency^29^.

### Physiological response during long-term proliferation

The accumulation of starch, soluble sugars, and soluble proteins plays a critical role in EC proliferation and SE development^51^. Starch, as a primary energy source, supports cell division and differentiation^57^. Starch content exhibited a declining trend with successive subcultures, consistent with findings in sugarcane^58^.

Soluble proteins and soluble sugars, essential for metabolic energy and osmotic balance. Studies on *Catharanthus roseus* and sugarcane have demonstrated that EC contains higher levels of soluble proteins and soluble sugars compared to non-embryogenic callus^58,59^. Similarly, positive correlations between soluble proteins content and SE yield have been reported in *Pinus thunbergia* and pear cultivars^33,60^. In this study, soluble proteins and soluble sugars contents, along with proliferation coefficient, initially increased but subsequently declined with successive subcultures. This trend highlights the critical role of these storage compounds in supporting EC proliferation and SE development.

Antioxidant enzymes, such as SOD, CAT, and POD, regulate oxidative stress by regulating intracellular reactive oxygen species levels^61^. In this study, the results of correlation and path analyses between physiological indicators and proliferation coefficient indicate that the proliferation coefficient of EC is mainly related to the content of storage substances (starch and soluble sugars) and the activities of redox-related enzymes (POD and CAT). This result aligns with findings from studies on *Pinus koraiensis*^38^ and *Pinus massoniana*^27^. Similar findings also have been observed in other tree species, in *Pinus pinaster*^62,63^ the accumulation of storage soluble proteins during the mid-maturation phase of SE induced by EC serves as a reliable marker for high-quality somatic embryos. In *Pinus massonian*^27^and in sugarcane^58^non-embryogenic callus exhibits higher levels of polyphenols and polyphenol oxidase activity, whereas callus that has lost its embryogenic potential is characterized by reduced levels of storage substances (soluble proteins, soluble sugars, and starch) and increased activities of POD and CAT.

This study established a stable long-term proliferation system for slash pine EC, while our study focused on physiological and proliferation metrics, genetic stability analysis (e.g., karyotyping, SSR markers, or flow cytometry) would be essential for future validation.

## Conclusion

This study established a stable long-term proliferation system for slash pine EC, with an optimal subculture interval of 14 days. The highest proliferation coefficient (7.65) was achieved using DCR medium supplemented with 1.0 mg/L 2,4-D and 1.0 mg/L 6-BA and embryogenic potential were maintained for up to three years. Under optimal conditions, additionally including 1.0 mg/L NAA, the maximum SE yield reached 143.25 per gram of EC. Regular medium replacement every 14 days ensured consistent proliferation and SE yield over 392 days of subculturing. Physiological analysis identified soluble sugars, starch contents, POD activity and CAT activity are key indicators of proliferation efficiency. This study initially overcame the difficulty of long-term subculture and loss of embryogenicity of embryonic callus in slash pine, achieving stable proliferation of embryonic callus (980 days) and maintenance of embryogenicity (352 days). And offering valuable insights into the physiological mechanisms underlying EC proliferation and embryogenic potential during somatic embryogenesis. This system has laid a technical foundation for the factory production of somatic embryogenesis in slash pine.

## Electronic supplementary material

Supplementary Material 1

Supplementary Material 2

## Data Availability

The datasets generated during and/or analyzed during the current study are available from the corresponding author on reasonable request.

## Abbreviations

PGRs: Plant growth regulators
EC: Embryogenic callus
SE: Somatic embryos
ESM: Embryonal suspensor mass
PEM: Pro-embryogenic mass
DCR: Douglas-fir Cotyledon Revised medium
2, 4-D: 2, 4-dichlorophenoxyacetic acid
6-BA: 6-benzylaminopurine
ABA: Abscisic Acid
NAA: 2-(1-Naphthyl) acetic acid
KT: Kinetin
BL: Brassinolide
SOD: Superoxide dismutase
POD: Peroxidase
CAT: Catalase
