# Supplementary material for: Long term proliferation and physiological response of embryogenic callus in Slash pine (Pinus Elliottii Engelm)
Source: Sci Rep. 2025 Jul 1;15:21327. doi: 10.1038/s41598-025-06436-5 (PMC12216556; doi:10.1038/s41598-025-06436-5)
Supplement: Supplementary file 1 — Supplementary Material 1 [file 41598_2025_6436_MOESM1_ESM.docx]

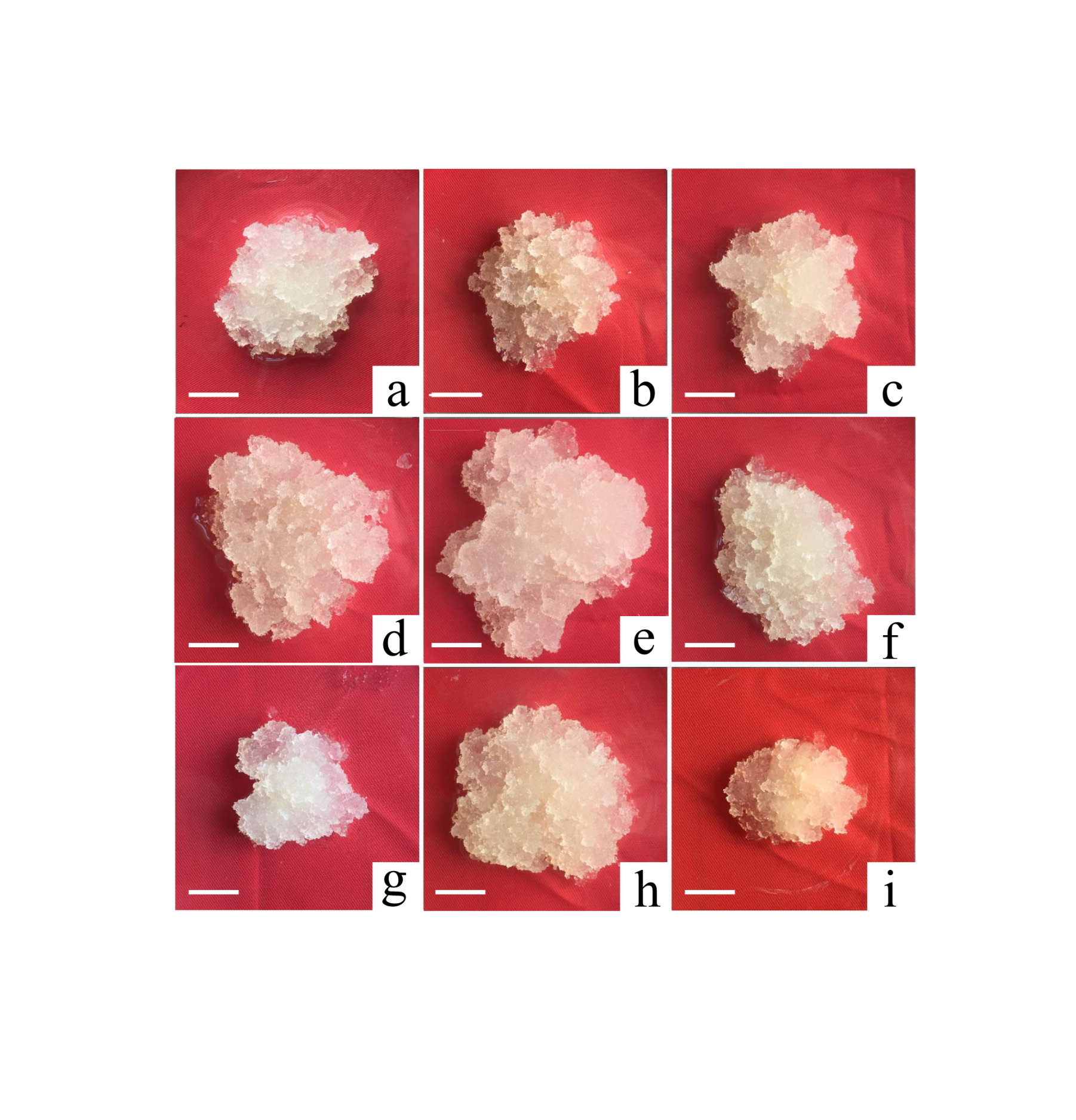


**Figuer S1.** Effect of PGRs concentration combinations on EC proliferation efficiency.

1. : Treatment 1, (b): Treatment 2, (c): Treatment 3, (d): Treatment 4, (e): Treatment 5, (f): Treatment 6, (g): Treatment 7, (h): Treatment 8, (i): Treatment 9. All bars= 1cm.
