# Supplementary material for: Long term proliferation and physiological response of embryogenic callus in Slash pine (Pinus Elliottii Engelm)
Source: Sci Rep. 2025 Jul 1;15:21327. doi: 10.1038/s41598-025-06436-5 (PMC12216556; doi:10.1038/s41598-025-06436-5)
Supplement: Supplementary file 2 — Supplementary Material 2 [file 41598_2025_6436_MOESM2_ESM.docx]

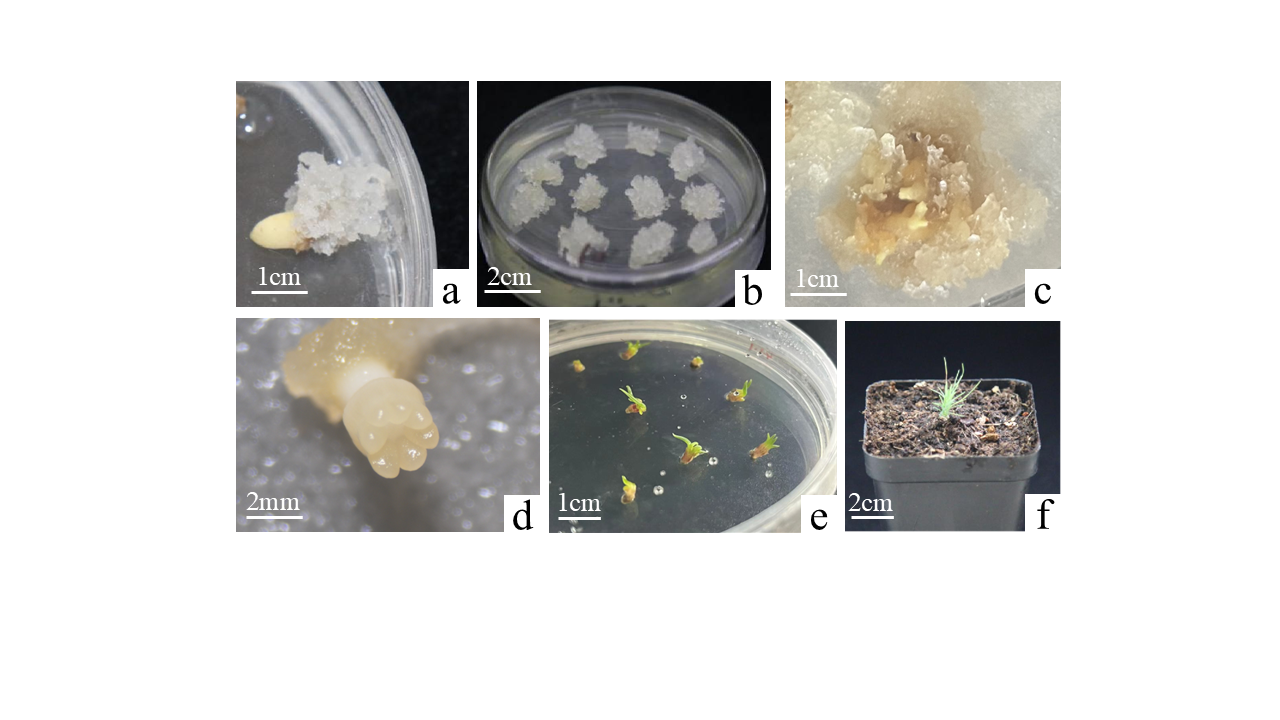


**Figure S2.** Somatic embryogenesis of slash pine. (a): EC on induction medium, (b): EC on proliferation medium, (c): SE induced on maturation medium, (d): SE, (e): somatic embryo-derived rootless seedlings, (f): Regenerated plants.
